# Synthesis, Structure, and Physicochemical Characteristics of Zn_1−x_Re_x_Cr_2_Se_4_ Single Crystals

**DOI:** 10.3390/ma16134565

**Published:** 2023-06-24

**Authors:** Izabela Jendrzejewska, Tadeusz Groń, Joachim Kusz, Zbigniew Stokłosa, Ewa Pietrasik, Tomasz Goryczka, Bogdan Sawicki, Jerzy Goraus, Josef Jampilek, Beata Witkowska-Kita

**Affiliations:** 1 Institute of Chemistry, University of Silesia in Katowice, 40-007 Katowice, Poland; ewa.pietrasik@us.edu.pl; 2 Institute of Physics, University of Silesia in Katowice, 40-007 Katowice, Poland; tadeusz.gron@us.edu.pl (T.G.); joachim.kusz@us.edu.pl (J.K.); bogdan.sawicki@us.edu.pl (B.S.); jerzy.goraus@us.edu.pl (J.G.); 3 Institute of Materials Science, University of Silesia in Katowice, 40-007 Katowice, Poland; zbigniew.stoklosa@us.edu.pl (Z.S.); tomasz.goryczka@us.edu.pl (T.G.); 4 Faculty of Natural Sciences, Comenius University, 842 15 Bratislava, Slovakia; 5 Katowice Branch, Research Network Łukasiewicz—Warsaw Institute of Technology, 00-661 Warsaw, Poland; b.witkowskakita@gmail.com

**Keywords:** spinel structure, chemical vapour transport, X-ray research, magnetic and electrical measurements, specific heat, thermal study

## Abstract

This study aimed to obtain and investigate ZnCr_2_Se_4_ single crystals doped with rhenium. The single crystals were obtained by applying chemical vapour transport. An X-ray study confirmed the cubic ($Fd\bar{3}m$) structure of the tested crystals. Thermal, magnetic, electrical, and specific heat measurements accurately determined the physicochemical characteristics, which revealed that the obtained single crystals are *p*-type semiconductors with antiferromagnetic order below the Néel temperature *T*_N_ = 21.7 K. The Debye temperature had a value of 295 K. The substitution of Re-paramagnetic ions, possessing a screened 5*d*-shell, in place of Zn-diamagnetic ions, caused an increase in the activation energy, Fermi energy, and Fermi temperature compared to the pure ZnCr_2_Se_4_. The boost of the *dc* magnetic field induced a shift of *T*_N_ towards lower temperatures and a spin fluctuation peak visible at H_dc_ = 40 and 50 kOe. The obtained single crystals are thermally stable up to 1100 °C.

## 1. Introduction

In the modern world, the synthesis of single crystals is a vast field of activity, encompassing theoretical aspects and device fabrication. Single crystals create the foundations of modern technology. Many types of crystals are needed for lasers, optical components, light-emitting diodes, electron emitters for electron microscopes, and countless other applications.

Materials with a spinel structure based on chromium and selenium (ACr_2_Se_4_, where A=Cu, Cd, and Zn) and doped with various elements are attractive for their magnetic, electrical, and thermal properties. They can be insulators, semiconductors, conductors, superconductors, ferrimagnets, ferromagnets, antiferromagnets, and Pauli paramagnets. In addition, these compounds are stable at high temperatures, so they can be applied in machines and devices operating at high temperatures. Doped spinel compounds, in which thermal conductivity exists, are possible for commercial application because thermoelectricity is a phenomenon that allows energy to be converted inside a solid. Thermoelectric cooling is the only environmentally friendly cooling used in power generators, computers, infrared detectors, and electronics and optoelectronics [1]. Doped chromite selenides have catalytic properties and could replace expensive silver catalysts in many chemical reactions [2]. In laser technology, the same compounds are used as materials that enable the use of solar energy [3,4].

The parent compound ZnCr_2_Se_4_, possessing semiconducting and antiferromagnetic (AFM) properties, belongs to the group of compounds crystallising in the spinel structure [5,6,7]. Because of the significant lattice parameter of ZnCr_2_Se_4_ (10.484 Å), an antiferromagnetic order below the Néel temperature *T*_N_ = 21 K, and the positive value of the paramagnetic Curie–Weiss temperature (*θ_CW_* = 115 K), the compounds based on doped ZnCr_2_Se_4_ are promising new materials with desired properties [8,9,10].

Numerous experimental and theoretical studies have been devoted to ZnCr_2_Se_4_ doped with various elements. The matrix was doped with *p*-elements (Al, Ga, In, Sn, and Pb [11,12,13,14,15,16,17,18]), *d*-elements (Cu, Mn, Ni, and Ta [19,20,21,22,23,24,25,26]), and *f*-elements (Dy, Gd, Ho, and Nd [27,28,29,30,31]).

The presence of an additional cation in the crystal lattice of ZnCr_2_Se_4_ caused significant phenomena, e.g., the re-entrant spin-glass, geometric spin-glass, and metal-insulator transition and heavy fermion properties [16,27,30,31]. The current article continues our earlier research, focusing on introducing *d*-elements to the crystal lattice of ZnCr_2_Se_4_ to obtain new materials with desired properties. This work presents the synthesis and physicochemical characteristics of ZnCr_2_Se_4_ single crystals doped with rhenium ions instead of zinc ions. The thermodynamic model, describing the growth of single crystals, has been elaborated using a computer program.

The determination of the Fermi energy and Fermi temperature from the diffusion component of the thermoelectric power has demonstrated the importance of this work. These phenomena have not been reported earlier in the literature.

## 2. Materials and Methods

### 2.1. Materials

To synthesise the ZnCr_2_Se_4_ single crystals doped with rhenium, the elements Zn, Re, Cr, and Se, and anhydrous CrCl_3_ (5N, Sigma Aldrich, Poznań, Poland) were applied.

### 2.2. Methods

#### 2.2.1. Chemical Vapour Transport (CVT)

The growth of ZnCr_2_Se_4_ single crystals doped with rhenium was carried out using chemical vapour transport (CVT). The CVT method is of great practical and cognitive importance. Crystallisation may occur at a temperature below the melting point. Thanks to this fact, it is possible to obtain structurally pure, low-temperature polymorphs.

Chemical vapour transport (CVT) is based on heterogeneous reversible reactions that can occur in the investigated system.

The order of magnitude of the equilibrium constants *K_p_* (*logK_p_* ≈ 0) regulates the transport ability of the reactions. The change of free energy Δ*G*° (close to zero) guarantees the process’s reversibility and ensures that significant amounts of products and substrates are in the equilibrium state. The value of Δ*G*° is calculated based on the formula below:

(1) $$\Delta G{}^{\circ}=-2.303RTlogK_{p}=\Delta H{}^{\circ}-T\Delta S{}^{\circ}$$

where: *R*—gas constant, *T*—absolute temperature, *K_p_*—equilibrium constant, Δ*G*°, Δ*H*°, and Δ*S*°—changes in the reaction’s free energy, enthalpy, and entropy. The set of transport reactions is chosen based on thermodynamic data. As a transport agent, volatile halides (e.g., NbCl_5_, AsCl_3_, CrCl_3_, and CdCl_2_) or chlorine are usually used [32,33,34]. The details describing the basis of the chemical vapour transport are presented in [35]. The HSC Chemistry v6 computer program was used for preparing the model of single-crystal growth [36].

#### 2.2.2. Physicochemical Characteristic

The physicochemical characteristics were achieved using various methods: (1) magnetic and electrical measurements; (2) X-ray study; (3) specific heat measurements; and (4) thermal analysis. Electrical conductivity, σ(T), of single crystals under study was measured along the [001] direction by the DC method using a Keithley 6517B Electrometer/High Resistance Meter (Keithley Instruments, LLC, Solon, OH, USA) and within the temperature range of 77–400 K. The crystal, whose surfaces at the edges of the octahedron were polished into a rectangular parallelepiped, was placed between copper electrodes and pressed mechanically. The Seebeck coefficient, *S(T)*, was measured within the temperature range of 100–400 K with the help of a Seebeck Effect Measurement System (MMR Technologies, Inc., San Jose, CA, USA). The electrical and thermal contact between the single crystal and electrodes was achieved by a silver lacquer mixture (Degussa Leitsilber 200) [9,20,21,22,23,24,25,26,27].

Dynamic magnetic susceptibility (*ac*) was measured at an internal oscillating magnetic field of Hac = 1 Oe with an internal frequency of *f* = 120 Hz. Magnetization isotherms were measured at 2, 4, 10, 20, 40, 60, and 300 K using a Quantum Design MPMS-XL-7AC SQUID magnetometer (Quantum Design, San Diego, CA, USA) in applied external fields up to 70 kOe. The details of research methods, conditions of measurements, and types of equipment used to prepare the physicochemical characteristic of Zn_1−x_Re_x_Cr_2_Se_4_ single crystals are described in Refs. [17,27,28,29,30,31,37]. The effective magnetic moment µ_eff_ was calculated using the equation presented in Refs. [38,39]. The effective number of Bohr magnetons p_eff_ was calculated from the equation:

(2) peff=2pCr2+ xpRe2,

where x is the content of rhenium ions in the sample, $p\;=\;g\sqrt{J\left(J\;+1\right)}$ [40] for Cr^3+^ (S = 3/2; L = 3; J = 3/2, g = 2/5, basic term ^4^F_3/2_; for g = 2, p_eff_ = 3.873 [40]) and Re^2+^ (S = 5/2; J = 5/2 for L = 5; g = 2/7, basic term ^6^S_5/2_; for L = 0 and g = 2, p_eff_ = 5.916 [41]) ions with 3*d*^3^ and 5*d*^5^ electronic configuration, respectively. The equations for the magnetic superexchange integrals *J*_1_ and *J*_2_ are presented in Ref. [42]. Specific heat *C*(*T*) was measured in the 2–300 K temperature range and in the external magnetic field up to 4 T using Quantum Design PPMS (Physical Properties Measurement System) with heat-capacity and -resistivity options.

Thermal measurements were conducted using a Labsys Evo (Setaram Inc., Cranbury, NJ, USA) apparatus. The measurements were carried out in the flowing high-purity Ar-atmosphere with a heating rate of 10 °C/min.

The scanning electron microscope JEM 6480 (JEOL USA, INC., Peabody, MA, USA) was applied with an energy-dispersive X-ray spectrometer (SEM/EDS) to determine the chemical composition.

The X-ray diffraction was conducted at 293(1) K. The data were collected using a Super Nova X-ray diffractometer (Agilnt, Oxfordshire, UK) with a microfocus X-ray tube, optimised multi-layer optics for Mo-Kα (λ = 0.71073 Å) radiation, and an Atlas CCD detector. Accurate cell parameters were determined and refined with CrysAlisPro software (version 1.171.37.35, Agilent Technologies, Wrocław, 2014). Also, the CrysAlisPro program was used to integrate the collected data. The spinel structure ($Fd\bar{3}m$) was refined using the SHELXL-2013 program [43,44]. All atoms were refined with anisotropic displacement parameters.

## 3. Results and Discussion

### 3.1. Thermodynamic Model of Chemical Vapour Transport in the ZnSe-Re-Se-CrCl_3_ System

The thermodynamic model is based on a set of hypothetical reactions that can occur in the ZnSe-Re-Se-CrCl_3_ system (a set of reactions is presented in Supplementary Materials). Anhydrous CrCl_3_, used as a transport agent in temperatures above 773 K, dissociates on CrCl_2_ and Cl_2_. The compound CrCl_2_ reacts with Cl_2,_ and CrCl_4_ forms. The partial pressure of Cl_2_ and CrCl_2_ are very low compared to that of CrCl_3_ and CrCl_4_. The partial pressure coefficient Q (Q = p(CrCl_3_)/p(CrCl_4_) is 20 at 830 K because, during the sublimation of CrCl_3_, the gas phase contains 5% CrCl_4_. In the reaction system, three transport agents co-exist. For this reason, the consideration of hypothetical reactions which could appear in the reaction system should consider reactions with CrCl_3_, CrCl_4,_ and Cl_2_ [45,46].

**Figure 1:**
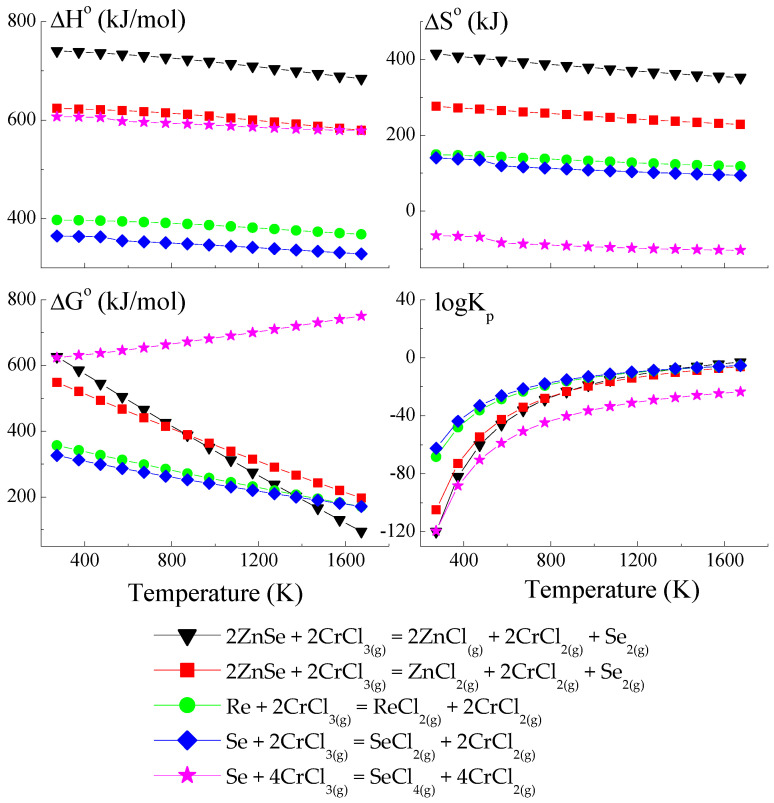
The dependence of the thermodynamic parameters vs. temperature *T* for the reactions with CrCl_3_ as a transport agent (theoretical calculation using HSC Chemistry v6 computer program [36]).

**Figure 2:**
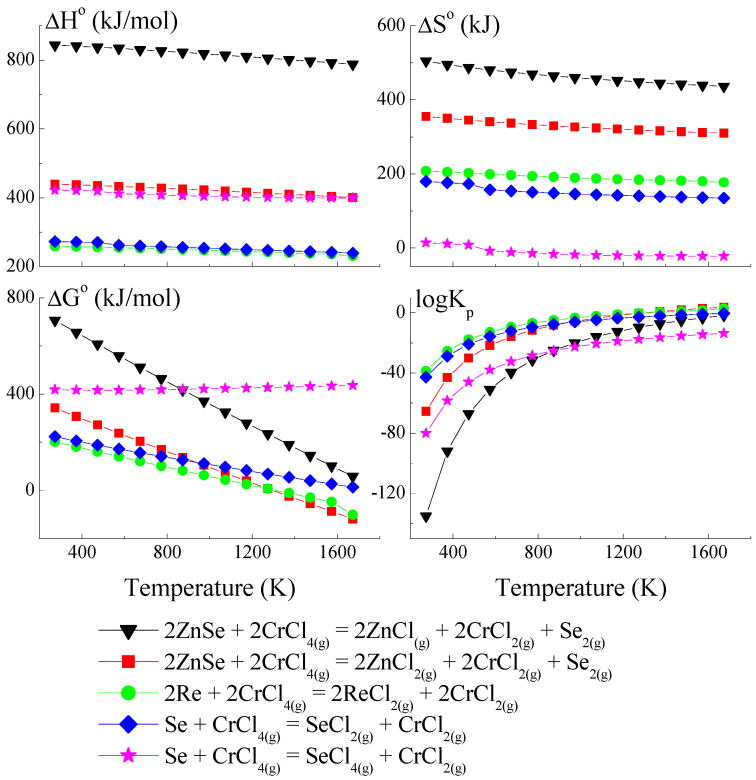
The dependence of the thermodynamic parameters vs. temperature *T* for reactions with CrCl_4_ as a transport agent (theoretical calculation using HSC Chemistry v6 computer program [36]).

**Figure 3:**
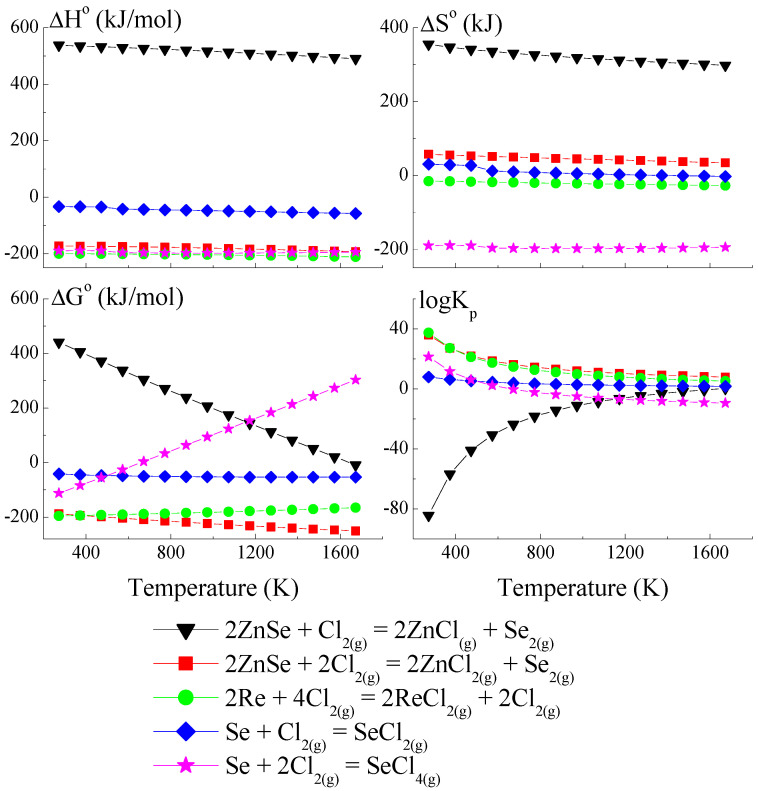
The dependence of the thermodynamic parameters vs. temperature *T* for reactions with Cl_2_ as a transport agent (theoretical calculation using HSC Chemistry v6 computer program [36]).

Because the compound ReSe does not exist, pure Re was utilised to calculate the crystal growth model for the ZnCr_2_Se_4_ single crystals doped with rhenium. The thermodynamic model of the chemical vapour transport in the ZnSe-Re-Se-CrCl_3_ system is presented in Figure 1, Figure 2 and Figure 3. The thermodynamic parameters (Δ*H*°, Δ*G*°, Δ*S*°, and *logK_p_*) were calculated using the HSC Chemistry 6 computer program [36].

The thermodynamic model of the chemical vapour transport in the ZnSe-Re-Se-CrCl_3_ system proved that the synchronous transport of ZnSe, Re, and Se would occur via gaseous CrCl_3_ and CrCl_4_ in the temperature range: 1000–1300 K. In this temperature range, *logKp* and Δ*G*° values are close to zero (Figure 1 and Figure 2), which indicates the proper conditions for chemical transport.

The prepared thermodynamic crystal growth model confirmed that for the ZnSe-Re-Se-CrCl_3_ system, the conditions for the simultaneous transport of ZnSe, Re, and Se are fulfilled.

### 3.2. Growth of ZnCr_2_Se_4_ Single Crystals Doped with Rhenium

The process of growth of the ZnCr_2_Se_4_ single crystals doped with rhenium was carried on in quartz-glass ampoules using a solid-state reaction in a high vacuum (10^−5^ mbarr). The experiments were carried out in ampoules with an outer diameter of 20 mm and a length of about 200 mm. The stoichiometric amounts of ZnSe, Re, and Se, weighed according to the reaction:

4(1 − x)ZnSe + 4xRe + 4xSe + 2CrCl_3_ = Zn_1−x_Re_x_Cr_x_Se_4_ + 3(1 − x)ZnCl_2_ + 3xReCl_2_ for x = 0.1–0.4.

Were introduced to the quartz-glass ampoule, sealed, and put into the two-zone pipe furnace.

Based on the thermodynamic model data, reaction conditions were selected: the dissolution zone 1143–1203 K, crystallisation zone 1103–1173 K, and temperature gradient 30–40 K (Table 1), according to Refs. [24,25,26,27,28,29,30,31,32]. The stoichiometric amounts of ZnSe, Re, Se, and CrCl_3_ placed in quartz ampoules were heated for 336 h and then cooled at about 50 degrees per hour. Based on the ideal gas equation of state

**Figure 4:**
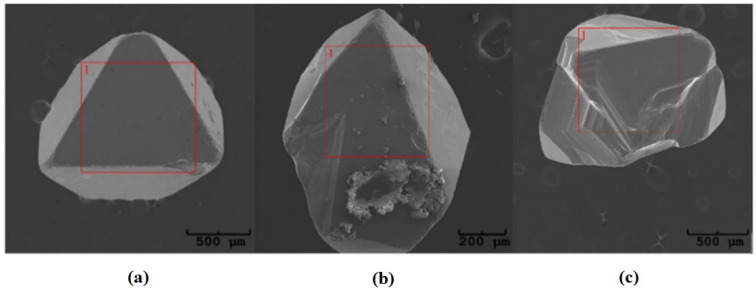
Examples of single crystals obtained in the ZnCr_2_Se_4_:Re system: (**a**) Zn_0.94_Re_0.06_Cr_2_Se_4_, (**b**) Zn_0.92_Re_0.08_Cr_2_Se_4_, and (**c**) Zn_0.88_Re_0.12_Cr_2_Se_4_.

(3) pV=nRT

where *p*—pressure, *V*—the volume of glass ampoule, *n*—the number of moles of transport agent, *R*—the universal gas constant, and *T*—temperature, it can be estimated that the average pressure in the quartz ampoule during the crystal growth. According to our calculations, the pressure inside the ampoule is about 0.3 MPa, which indicates that the substrates were transferred into the gas phase by diffusion [32,33]. The obtained ZnCr_2_Se_4_:Re single crystals are shown in Figure 4.

### 3.3. Chemical Composition

Each tested single crystal was measured at 20 different locations. The measuring area was approximately 50 × 30 μm^2^ (Figure 4). The results are presented in Table 1.

### 3.4. Structural Study

**Figure 5:**
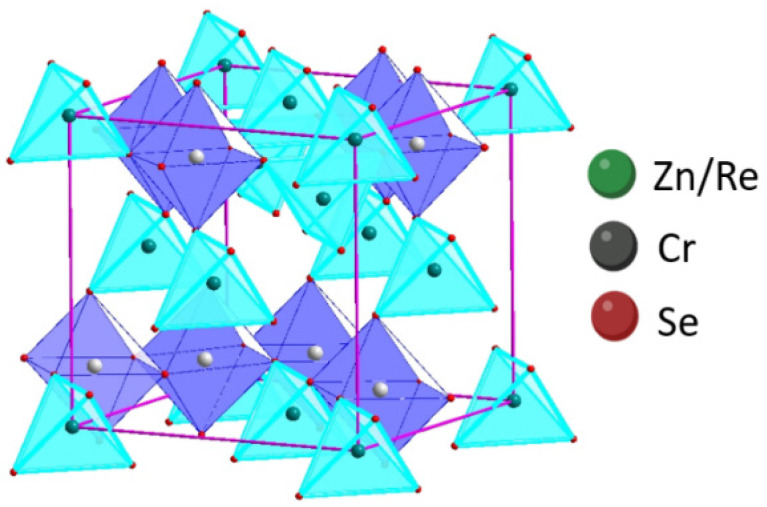
Projection of cubic structure of Zn_1−x_Re_x_Cr_2_Se_4_ crystals.

The structural parameters were calculated based on the procedure depicted in Refs. [18,31]. The results showed that the Re ions share the tetrahedral positions with Zn-cations in every fifth crystal. The structural study confirmed that the obtained samples crystallise in the cubic system (SG $Fd\bar{3}m$). The formula describing cation distribution in the obtained ZnCr_2_Se_4_ single crystals doped with rhenium is **Zn_1−x_Re_x_Cr_2_Se_4_** (Figure 5).

**Figure 6:**
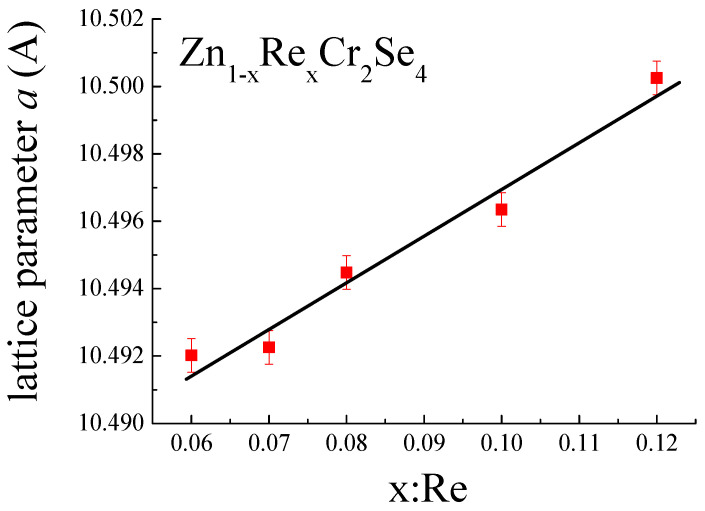
Change in lattice parameter *a* as function of rhenium quantity.

The presence of Re ions in ZnCr_2_Se_4_ is confirmed by an increase in lattice parameters of the unit cell (Table 2, Figure 6), by the difference of ionic and covalent radii (rZn i=0.60 Å, rRei=0.96 Å, RZnC=1.33 Å, RReC=1.41 Å, where *r* indices ionic radius, and R indices covalent radius [47,48]). The linear dependence of lattice parameters indicates that Vegard’s rule is observed in Zn_1−x_Re_x_Cr_2_Se_4_.

The details about other structural parameters (CIF files, all measurement parameters, values of the parameter *u*, atomic coordinates, equivalent isotropic displacement parameters, interatomic distances, and bond angles) of the Zn_1−x_Re_x_Cr_2_Se_4_ single crystals are presented in the Supplementary Materials.

### 3.5. Electrical Studies

The activation energy *E*_a_ was calculated according to the formula:

(4) $$\sigma \;={\;\sigma }_{0}\exp\left(-\frac{E_{a}}{\mathrm{kT}}\right),$$

where *k* is the Boltzmann constant and σ_0_ is the reference conductivity.

**Figure 7:**
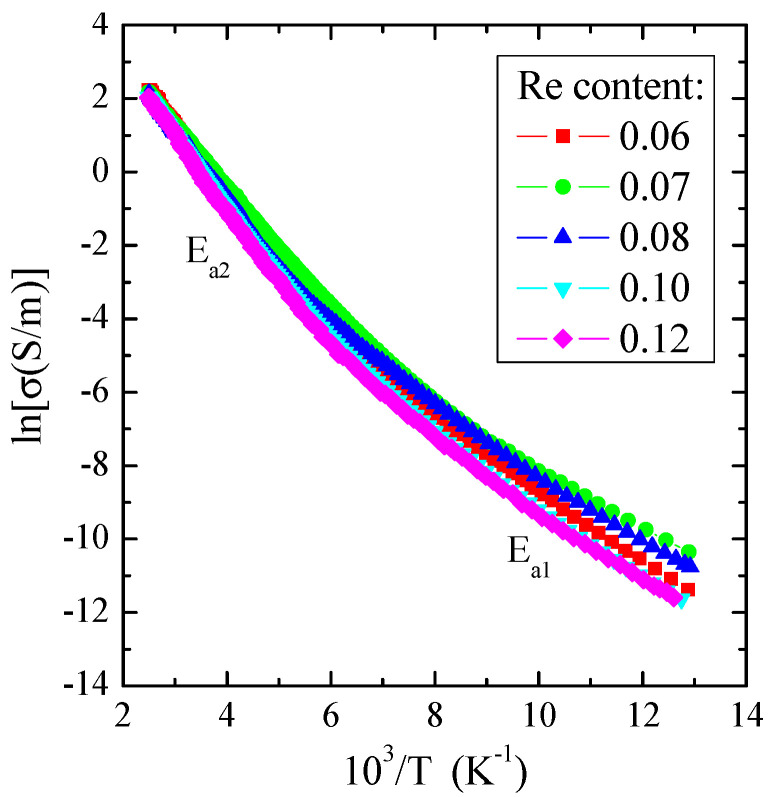
Electrical conductivity (lnσ) vs. reciprocal temperature (10^3^/T) for Zn_1−x_Re_x_Cr_2_Se_4_ single crystals.

As illustrated in Figure 7, the electrical conductivity for measured crystals revealed two areas: the external area in the narrow temperature range of 77–130 K, in which the weak thermal activation of *E*_a1_~0.08 eV is observed, and the internal area in the temperature range of 200–400 K with stronger thermal activation of *E*_a2_~0.16 eV (Table 3). In the region of stronger activation, the value of electrical conductivity at 400 K is about 9 S/m, a typical value for a spinel matrix with an energy gap of 1.28 eV at room temperature [49]. The admixture of rhenium generally enhances the thermal activation of current carriers in the internal region compared to ZnCr_2_Se_4_, for which *E*_a_ is about 1.35 eV [13] (Table 3). Similar behaviour was found for the ZnCr_2_Se_4_ crystals doped with elements 3*d* [14], 5*d* [19,24], and 4*f* [29,31].

**Figure 8:**
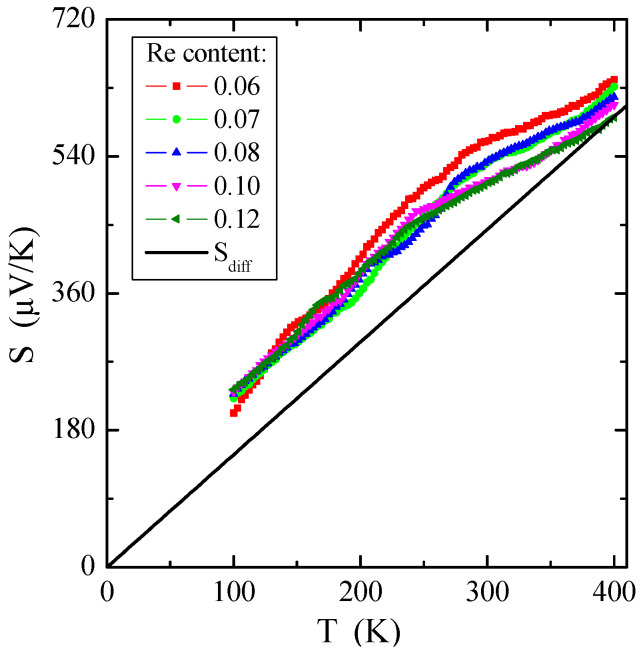
Thermoelectric power *S* vs. temperature T of Zn_1−x_Re_x_Cr_2_Se_4_ single crystals. *S_diff_* is the diffusion component of thermopower (marked with a solid line).

Figure 8 demonstrates the dependence of thermoelectric power on temperature *S*(*T*). On the whole, the thermopower in conventional metals is composed of two various components: (1) a diffusion component (*S_diff_*), which is proportional to temperature according to the Mott formula at higher temperatures [50], and a phonon drag component (*S_ph_*), which is more complex. The *S_ph_* contribution comes from transferring phonon momentum to the electron gas. Both components drop at low temperatures, such as *T*^3^ below θ_D_/10, when phonons freeze out (θ_D_ is the Debye temperature), and at high temperatures, such as *T*^−1^ above approximately θ_D_/2, when the phonon’s excess momentum is limited by anharmonic phonon–phonon scattering [51]. A Debye temperature of Zn_1−x_Re_x_Cr_2_Se_4_, obtained from specific heat measurement, has a value of 295 K. Therefore, the peak of the phonon drag component of thermoelectric power should be in the temperature range of 30–140 K and is not visible in Figure 8. The diffusion share *S_diff_* is a direct application of the Boltzmann transport equation [50], described by the formula:

(5) $$S_{diff}=\frac{\pi^{2}k^{2}}{eE_{F}}T=aT,\;$$

where *e* is the elementary charge, *E_F_* is the Fermi energy, and *a* is an empirical slope. Using Equation (5), the Fermi energy, *E_F_*, can be determined by the formula:

(6) $$E_{F}=\frac{\pi^{2}k^{2}}{ea}.\;$$

The experimental dependence of *S_diff_* on temperature is evident in Figure 8 by solid lines. Based on Equation (6), it is possible to estimate the Fermi energy *E_F_* and the Fermi temperature *T_F_* (defined as *E_F_*/*k*), knowing the experimental value of the slope of thermopower for every single crystal. The values of *E_F_* and *T_F_* are given in Table 3. Comparing metals, e.g., for pure copper, *E_F_* = 7 eV and *T_F_* = 8.19 × 10^4^ K [52], and non-metallic conductors, e.g., for Cu_1−x_Ga_x_Cr_2_Se_4_ single crystals, *E_F_*~0.3 eV and *T_F_*~3 × 10^3^ K [53], it can be concluded that the Fermi energy *E_F_* has smaller values for the tested crystals. It bears out that the Fermi level is near the valence-band border, and the shallow acceptor level is just above the valence band. The source of the observed low *p*-type electrical conductivity, which is more thermally activated above room temperature, could be cationic vacancies in the spinel structure. Structural defects seem to always exist at thermal equilibrium in the crystal lattice, even in perfect samples.

**Figure 9:**
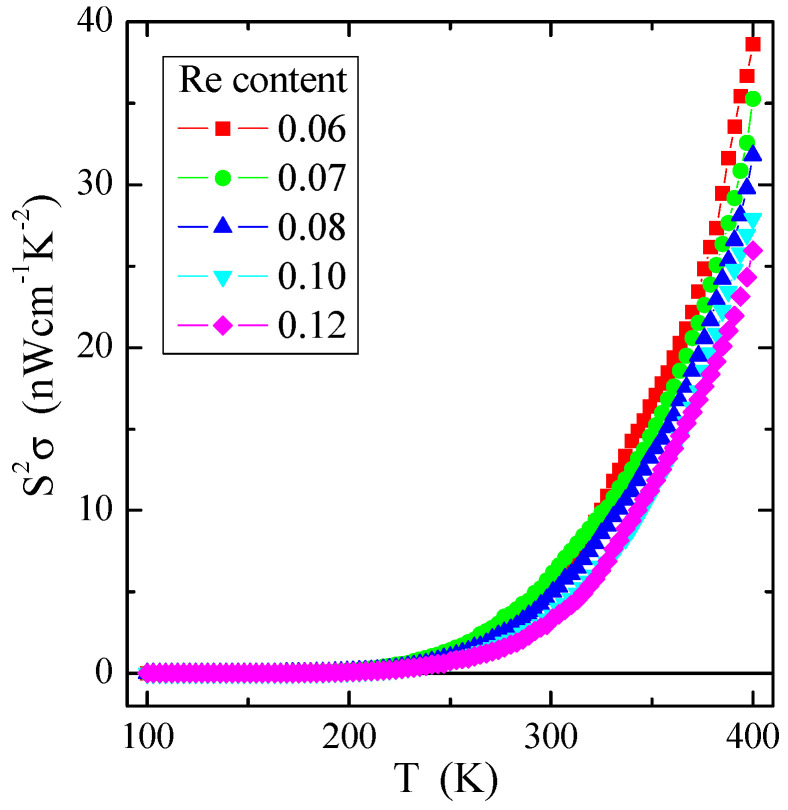
Power factor S^2^σ vs. temperature T of Zn_1−x_Re_x_Cr_2_Se_4_ single crystals.

As evident from Figure 9, the power factor S^2^σ of the investigated spinel semiconductors has a small value of several tens of nW/(cmK^2^), e.g., compared to the value of 0.1 µW/(cmK^2^) for the non-metallic spinel conductor CuCr_2_Se_4_:Ga [1]. The value of S^2^σ substantially increases with increasing temperature, i.e., in the internal area already above 250 K for all investigated samples with rhenium. Similar behaviour of the power factor as a function of temperature, but, on a somewhat smaller scale, was observed in the somewhat conductive molybdate-tungstate ceramics doped with Ga^3+^ and Co^2+^ [54], as well as Nd^3+^ and Mn^2+^ [55]. The above studies indicate that, regardless of the chemical bond type, the appropriate admixture influences the power factor, increasing the thermal activation of electric current carriers.

### 3.6. Magnetic Properties

**Figure 10:**
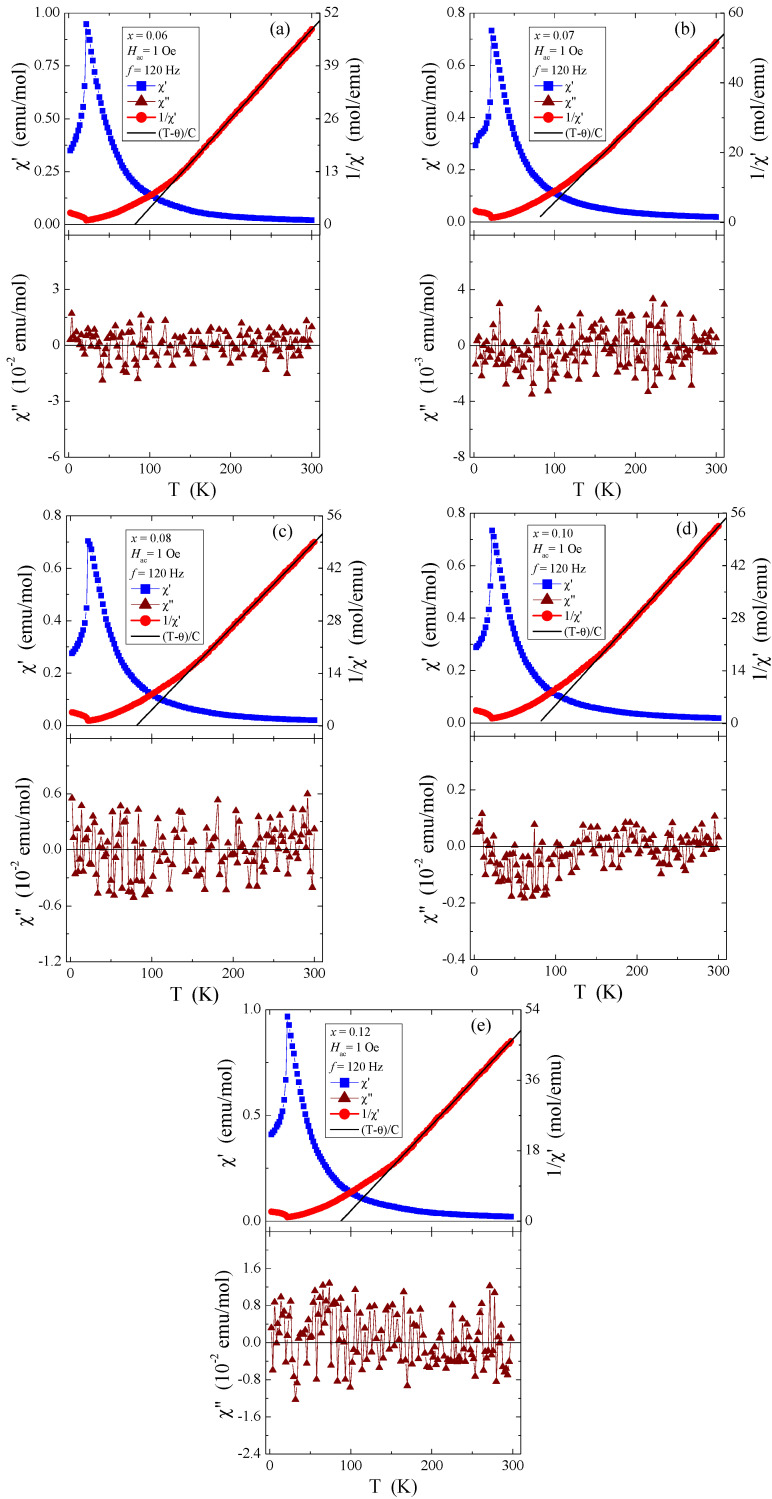
The real (χ′) and imaginary (χ″) components of ac magnetic susceptibility and 1/χ′ vs. temperature *T* of Zn_1−x_Re_x_Cr_2_Se_4_ single crystals: (**a**) Zn_0.94_Re_0.06_Cr_2.0_Se_4.0_, (**b**) Zn_0.93_Re_0.07_Cr_2.0_Se_4.0_, (**c**) Zn_0.92_Re_0.08_Cr_2.0_Se_4.0_, (**d**) Zn_0.90_Re_0.10_Cr_2.0_Se_4.0_, (**e**) Zn_0.88_Re_0.12_Cr_2.0_Se_4.0_.

The measurement data in Figure 10a–e, recorded in the internal oscillating magnetic field *H_ac_* = 1 Oe with the internal frequency *f* = 120 Hz and with zero external static magnetic field, suggest that the dependence of both magnetic susceptibility components (real (χ′) and imaginary (χ″)) on temperature indicates the AFM behaviour below the Néel temperature *T*_N_ = 21.7 K and at positive Curie–Weiss temperatures (θ) of about 80 K. These values are typical for short-range FM interactions, which are independent of the quantity of rhenium in the studied samples. On the other hand, the oscillations of the imaginary component of the *ac* magnetic susceptibility, χ″, visible around the value of zero (Figure 10), suggest the lack of energy losses caused, among others, by spin reorientation or rotation of the domain walls.

**Figure 11:**
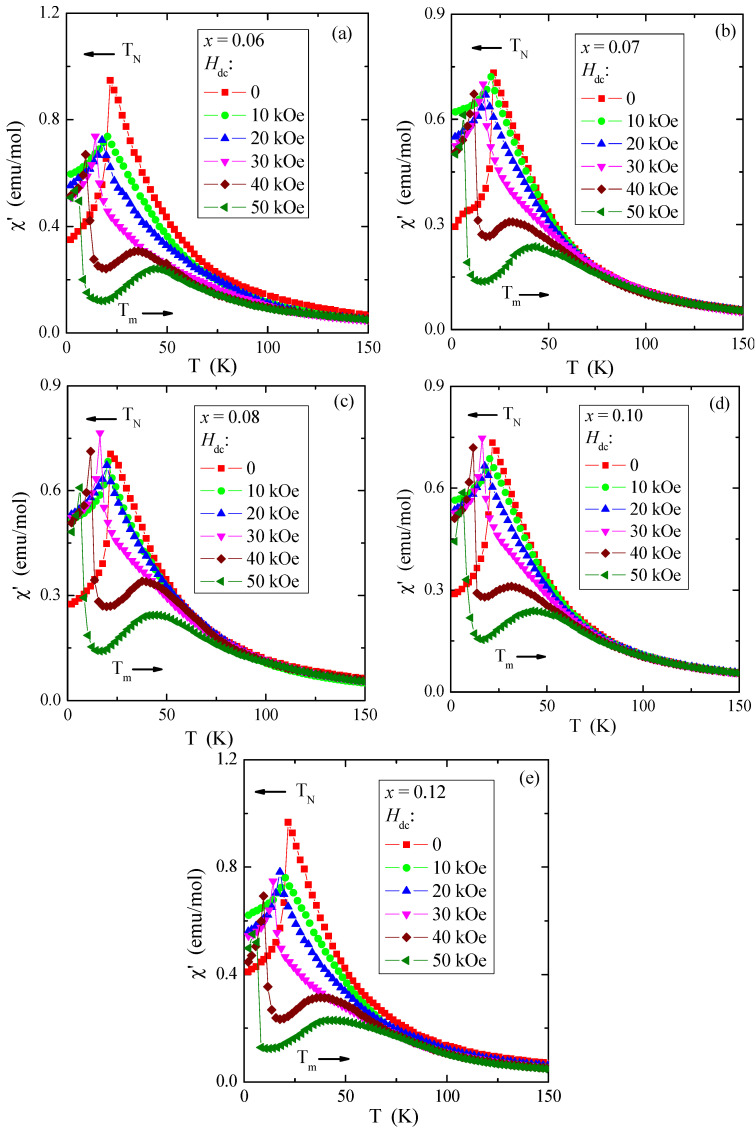
Ac magnetic susceptibility χ′ vs. temperature *T* recorded at internal oscillating magnetic field *H_ac_* = 1 Oe with internal frequency *f* = 120 Hz for Zn_1−x_Re_x_Cr_2_Se_4_ single crystals. Horizontal arrows indicate the shift of *T*_N_ and *T_m_* with increasing magnetic field *H_dc_*: (**a**) Zn_0.94_Re_0.06_Cr_2.0_Se_4.0_, (**b**) Zn_0.93_Re_0.07_Cr_2.0_Se_4.0_, (**c**) Zn_0.92_Re_0.08_Cr_2.0_Se_4.0_, (**d**) Zn_0.90_Re_0.10_Cr_2.0_Se_4.0_, (**e**) Zn_0.88_Re_0.12_Cr_2.0_Se_4.0_.

The determined values of θ, presented in Table 4, are much lower than the literature data shown in Refs. [5,6] and slightly lower than in [8], where θ is 115 K and 90 K, respectively. In contrast, the determined T_N_ values for all tested crystals are the same and close to those of pure ZnCr_2_Se_4_ [5,6,9]. Long-range AFM interactions are less sensitive to doping with paramagnetic ions, whose 4*f* and 5*d* orbitals are intensely screened. On the other hand, FM short-range interactions are more sensitive to the local spin ordering visible in the oscillatory character of the values of both the paramagnetic Curie–Weiss temperature and the *J_1_* and *J_2_* superexchange integrals for the first two coordination spheres (Table 4). The values of the effective magnetic moment (μ_eff_) are substantially similar to the values of the effective number of Bohr magnetons (p_eff_) for the electron configuration of rhenium 5*d*^5^ and the base term ^6^S_5/2_. It may mean that the orbital magnetic contribution has been quenched and the contribution to the magnetic moment comes solely from the spin. The temperature dependences of *ac* magnetic susceptibility χ′, recorded in the internal oscillating magnetic field *H_ac_* = 1 Oe with the internal frequency *f* = 120 Hz and taken at external static magnetic fields *H_dc_* = 0, 10, 20, 30, 40, and 50 kOe, are depicted in Figure 11a–e. This figure shows that *T*_N_ is shifted toward lower temperatures and *T_m_* is shifted toward higher ones. A strong magnetic field diminishes the AFM order and enlarges the FM order.

Additionally, the Curie constant *C* and the effective moment µ_eff_ are slightly higher than the chromium ion value per molecule. It is confirmed that the rhenium ions influence the magnetic moment. For *H_dc_* = 50 kOe, the *J*_1_ superexchange integral for the first coordination sphere changes the sign from negative to positive, while the *J*_2_ integral remains positive (not shown here), as in Zn_1−x_Pb_x_Cr_2_Se_4_ [18].

It corroborates that the short-range FM interaction expands through the whole temperature range. The (χ′(T) curves above *T*_N_, in the paramagnetic region, illustrate character istic broad peaks at *T_m_* = 31–35 and 42–45 K in the fields *H_dc_* = 40 and 50 kOe, respectively. These broad peaks of *ac* magnetic susceptibility may be caused by the spin fluctuations that appear due to a static magnetic field strengthening short-range FM interactions. The thermal energy *kT* opposes this phenomenon. Similar peaks were observed in the ZnCr_2_Se_4_ crystals doped with Al [56], Ce, Ga, In [57], and Pb [18].

**Figure 12:**
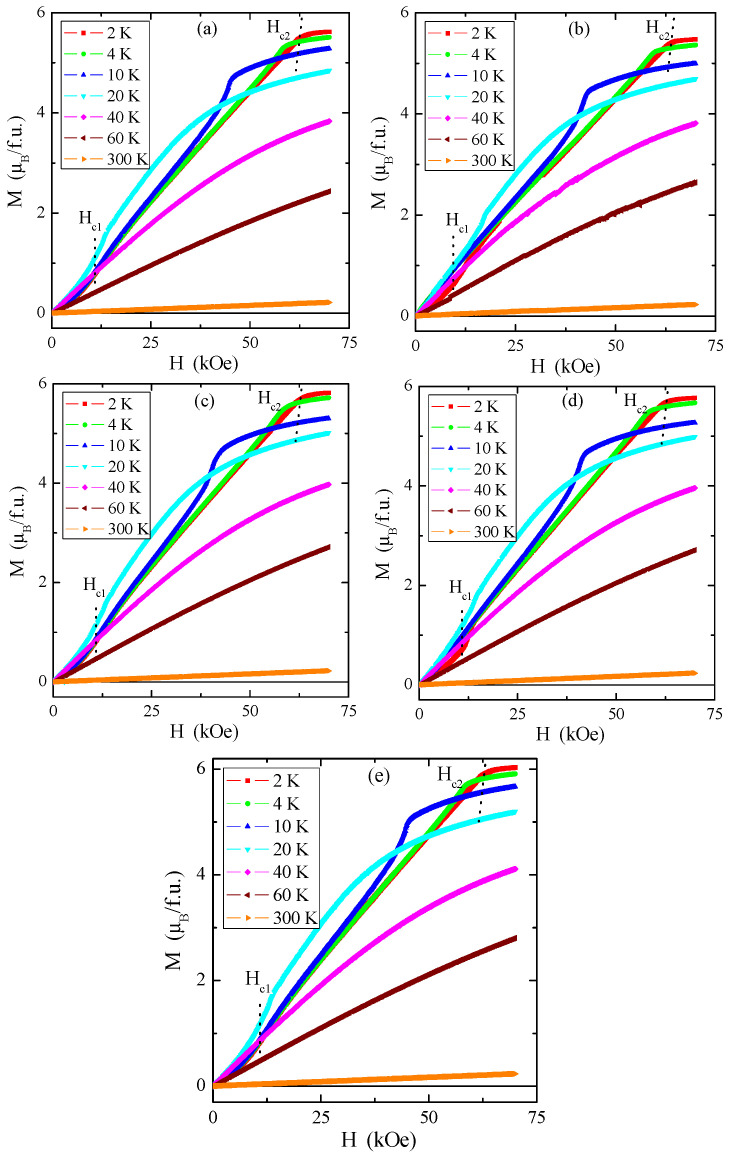
Magnetization *M* vs. magnetic field *H* for Zn_1−x_Re_x_Cr_2_Se_4_ single crystals: (**a**) Zn_0.94_Re_0.06_Cr_2.0_Se_4.0_, (**b**) Zn_0.93_Re_0.07_Cr_2.0_Se_4.0_, (**c**) Zn_0.92_Re_0.08_Cr_2.0_Se_4.0_, (**d**) Zn_0.90_Re_0.10_Cr_2.0_Se_4.0_, (**e**) Zn_0.88_Re_0.12_Cr_2.0_Se_4.0_. The first (*H_c_*_1_) and second (*H_c_*_2_) critical magnetic fields registered at 2 K are marked with dotted lines.

Magnetic isotherms in Figure 12a–e indicate that the magnetic saturation value of the tested single crystals is close to 6 μ_B_/f.u., similar to pure ZnCr_2_Se_4_ [4].

The first critical field *H_c_*_1_, i.e., metamagnetic transition, is connected with the transition from the helical to the conical phase, and the second critical field *H_c_*_2_ is related to the change of the spiral spin order to the ferromagnetic phase. The critical fields *H_c_*_1_ (a value of approx. 12 kOe) and *H_c_*_2_ (a value of approx. 61 kOe) insignificantly depend on the rhenium quantity in the sample. Comparison with pure ZnCr_2_Se_4_ revealed that *H_c_*_1_ has a somewhat higher value and *H_c_*_2_ has a slightly lower value (Table 4) [8]. The hysteresis loops have zero-field coercivity and zero remanences.

### 3.7. Specific Heat Measurements

**Figure 13:**
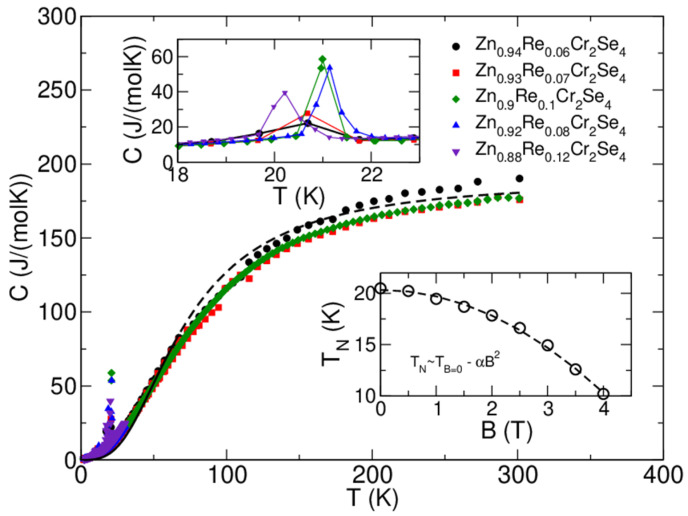
Specific heat, *C*, as a function of temperature *T*, measured for Zn_1−x_Re_x_Cr_2_Se_4_ single crystals at zero magnetic field (central figure). The dashed lines indicate that the Debye model fit experimental data for T > 40 K and the Zn_0.94_Re_0.06_Cr_2_Se_4_ sample (black-filled circles). The upper inset shows how the magnetic peak is affected by Re substitution. The lower inset shows how the magnetic field affects the magnetic transition temperature for the Zn_0.93_Re_0.07_Cr_2_Se_4_ sample. Experimental data is presented here with open circles, and the quadratic fit is given with the dashed line. Note that the temperature scales on the lower and upper insets are different. Hence, the peaks in the upper inset appear much broader than in the lower inset or the central figure.

The results of specific heat measurements are shown in Figure 13. Moreover, the dashed line indicates a Debye model fit for *T* > 35 K and sample composition Zn_0.95_Re_0.06_Cr_2_Se_4_ with the following fit parameters: number of atoms *n_D_*~7.59 and Debye temperature θ_D_~295 K [30]. The number of atoms fits the stoichiometry quite reasonably, and the pattern of the heat-capacity curves is similar for all the samples (which is expected, due to their composition). Therefore, the Debye temperature is also similar. The upper inset shows the magnetic peak at *T*_N_~21 K for various stoichiometries. The position of the peak does not shift monotonously with Re concentration. The difference in ordering temperature, as seen from the heat-capacity measurement, is below~1 K. Taking into account limitations of the pulse-measurement technique employed in the PPMS instrument, we can say that Re substitution does not change the ordering temperature in an observable and consistent way. The lower inset shows the position of the magnetic peak for the Zn_0.95_Re_0.06_Cr_2_Se_4_ sample in magnetic fields up to 4 T. We see that the increasing field shifts the peak to lower temperatures, confirming the antiferromagnetic ordering. The dependence can be very well fitted with the quadratic formula T_N_ = T(B = 0T) − α^2^ (α = 0.63 and T(B = 0) = 20.34 K).

### 3.8. Thermal Study

**Figure 14:**
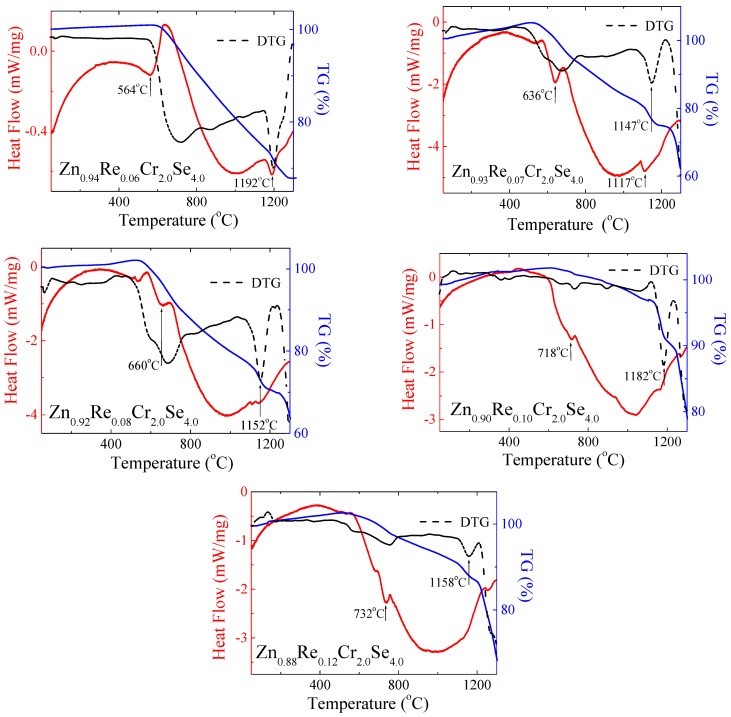
DSC/TG curves for Zn_1−x_Re_x_Cr_2_Se_4_ single crystals (DTG—Derivative Thermogravimetry).

The thermal study results are presented in Figure 14 and Table 5. The shape of DSC curves revealed that the obtained Zn_1−x_Re_x_Cr_2_Se_4_ single crystals are stable up to above 500 °C. For the smaller amount of rhenium (x = 0.06; 0.07), the first endothermic peaks appear at 564 °C and 636 °C, respectively. For both compounds, the second endothermic peak is visible above 1100 °C (Figure 12). With increasing rhenium, the endothermic peaks are shifted towards higher temperatures, and the second endothermic peak disappears. This phenomenon can be associated with rhenium ions in the crystal lattice of ZnCr_2_Se_4_.

Re^2+^ ions have a bigger ionic radius (0.96 Å) than Zn^2+^ ions (0.60 Å), which can influence the stability of the crystal lattice. Small changes in the sample mass are observed with the endothermic peaks. It may indicate melting and evaporation processes, which can occur in the system during heating. However, it is worth highlighting that the observed changes are insignificant and suggest the thermal resistance of obtained crystals. The mass loss is observed with increasing temperature on the DTG curve. The extensive mass loss is observed above 1100 °C.

## 4. Conclusions

We have presented a new family of Zn_1−x_Re_x_Cr_2_Se_4_ single crystals. These single crystals have been obtained using the chemical vapour transport (CVT) technique. The conditions of the crystal growth process were refined using thermodynamic calculations. SEM and XRD studies indicated that the obtained single crystals are chemically pure and crystallised in the cubic system (SG: $Fd\bar{3}m$), which aligns with the spinel structure. Thermal measurements confirmed the thermal stability of single crystals at temperatures up to 1100 °C. Increasing the external magnetic field shifts *T*_N_ and the specific heat peak towards lower temperatures, while the values of *T_m_*—towards higher temperatures. A significant weakness of long-range AFM interactions is evidenced in the reduction of the superexchange integral *J*_1_ for the first coordination sphere and spin fluctuations in the paramagnetic region. The dependence of magnetisation on the magnetic field showed two untypical phenomena below *T*_N_. These phenomena occurred at critical fields *H_c_*_1_ = 12 and *H*_c2_ = 57 kOe. It is correlated well with the change of the sign of the *J*_1_ integral from negative to positive at *H_dc_* = 50 kOe, which is caused by the short-range FM interaction extending through the whole temperature range. Calculations of the Fermi energy (*E_F_*) and the Fermi temperature (*T_F_*) derived from the diffusive component of thermoelectric power revealed a slight increase in *E_F_* and *T_F_* with increasing rhenium content, indicating shallow acceptor levels above the valence band. A substantial increase in the thermoelectric power factor *S^2^σ* in the internal region above 250 K was observed for all samples containing rhenium.

Based on our investigations, we can conclude that obtained results provided compelling evidence that the materials found on the doped ZnCr_2_Se_4_ compound could be appropriately implemented in a broad spectrum of new technological areas, e.g., as thermomagnetic and thermoelectric materials in electronic devices. Our results encourage future studies and should be explored on this type of material.

## Figures and Tables

**Table 1:** Conditions of growth and chemical composition of ZnCr_2_Se_4_:Re single crystals.

N°	Amounts of Substrates (mmol)		*T*_d_ (K)	*T*_c_ (K)	Δ*T* (K)	% Weight				Chemical Formula
	n_ZnSe_	n*_ReSe_				Zn	Re	Cr	Se	
(1)	14.4	1.6	1173	1143	30	12.29 ± 0.19	2.02 ± 0.21	19.68 ± 0.36	66.01 ± 0.24	Zn_0.94_Re_0.06_Cr_2.0_Se_4.0_
(2)	12.8	3.2	1143	1103	40	12.07 ± 0.21	2.59 ± 0.36	19.79 ± 0.34	65.55 ± 0.28	Zn_0.93_Re_0.07_Cr_2.0_Se_4.0_
(3)	11.2	4.8	1203	1163	40	12.20 ± 0.23	3.12 ± 0.29	20.77 ± 0.35	63.91 ± 0.23	Zn_0.92_Re_0.08_Cr_2.0_Se_4.0_
(4)	9.6	6.4	1203	1173	30	11.57 ± 0.18	3.78 ± 0.24	20.99 ± 0.39	63.65 ± 0.21	Zn_0.90_Re_0.10_Cr_2.0_Se_4.0_
(5)	9.6	6.4	1193	1153	40	11.37 ± 0.18	4.21 ± 0.24	21.03 ± 0.39	63.39 ± 0.21	Zn_0.88_Re_0.12_Cr_2.0_Se_4.0_

*T*_d_ is the temperature of the dissolution zone, *T*_c_ is the temperature of the crystallisation zone, and Δ*T* is the temperature difference between the dissolution and crystallisation zones. * the theoretical (calculated) amount of ReSe.

**Table 2:** Structural parameters of Zn_1−x_Re_x_Cr_2_Se_4_ single crystals.

x	Lattice Parameter (Å)	Volume (Å^3^)	Density Calc. (Mg/m^3^)	Absorption Coeff. (mm^−1^)	Goodness of Fit on F^2^	R Parameters	
						R_1_	wR_2_
0.06	10.49212(16)	1155.02 (5)	5.664	33.879	1.176	0.0236	0.071
0.07	10.49206(19)	1155.00 (6)	5.678	34.048	1.720	0.0245	0.0760
0.08	10.49448(14)	1155.80 (1)	5.688	34.192	1.182	0.0266	0.0882
0.10	10.49635(14)	1156.42 (2)	5.713	34.509	1.212	0.0326	0.1077
0.12	10.50025(15)	1157.71 (1)	5.734	34.806	1.2690	0.0413	0.1259

**Table 3:** Electrical parameters of Zn_1−x_Re_x_Cr_2_Se_4_ single crystals.

Chemical Formula	*a* (µV/K^2^)	*E*_F_ (eV)	*T*_F_ (K)	*E*_a__1_ (eV)	*E*_a__2_ (eV)
Zn_0.94_Re_0.06_Cr_2_Se_4_	1.60	0.045	531	0.173	0.084
Zn_0.93_Re_0.07_Cr_2_Se_4_	1.58	0.046	534	0.146	0.072
Zn_0.92_Re_0.08_Cr_2_Se_4_	1.55	0.047	545	0.162	0.078
Zn_0.90_Re_0.10_Cr_2_Se_4_	1.52	0.048	557	0.172	0.083
Zn_0.88_Re_0.12_Cr_2_Se_4_	1.48	0.050	580	0.176	0.082

*a* is the slope of the linear *S_diff_*(T) diffusion function of thermopower; *E*_F_ is the Fermi energy; *T*_F_ is the Fermi temperature; *E*_a1_ and *E*_a2_ are the activation energies in the intrinsic and extrinsic regions, respectively.

**Table 4:** Magnetic parameters of Zn_1−x_Re_x_Cr_2_Se_4_ single crystals recorded in the internal oscillating magnetic field *H*_ac_ = 1 Oe with the internal frequency *f* = 120 Hz and with zero external static magnetic field.

x	C (emu·K/mol)	T_N_ (K)	θ (K)	µ_eff_ (µ_B_/f.u.)	p_eff_	M_s(2K)_ (µ_B_/f.u.)	J_1_ (K)	J_2_ (K)	H_c1_ (kOe)	H_c2_ (kOe)	E_a_ (eV)
0	4.082	21	90	5.714	5.477	6.0	−1.65	1.28	10.0	65.0	0.135
0.06	4.554	21.7	81.5	6.035	5.666	5.62	−1.90	1.22	11.4	61.2	-
0.07	4.365	21.7	74.7	5.901	5.697	5.48	−2.01	1.17	12.3	62.5	-
0.08	4.478	21.7	80.8	5.985	5.727	5.82	−1.91	1.22	11.1	61.0	-
0.10	4.212	21.7	79.2	5.804	5.788	5.77	−1.94	1.20	12.9	60.0	-
0.12	4.565	21.7	87.7	6.042	5.849	6.03	−1.79	1.27	12.4	61.8	-

C is the Curie constant, T_N_, θ, are the Néel and Curie–Weiss temperatures, respectively; µ_eff_ is the effective magnetic moment; p_eff_ is the effective number of Bohr magnetons; M_s(2K)_ is a magnetization at 2K; J_1_ and J_2_ are the superexchange integrals for the first two coordination spheres; H_c1_ and H_c2_ are the critical fields measured at the static magnetic field up to 70 kOe; and E_a_ is the energy activation at 300 K [12]. Experimental data for ZnCr_2_Se_4_ were taken from refs. [5,6,9,13] for comparison.

**Table 5:** Parameters determined from DSC/TG analysis for Zn_1−x_Re_x_Cr_2_Se_4_ single crystals.

Re Content	Weight Loss (%)	Onset (^o^)	Endset (^o^)	Peak Minimum (^o^)	Peak Height (mW)	Peak Area (J)	Enthalpy (J/g)
0.0 *	35	735	771	755	6.47	1.09	236
0.6	32	359 1164	629 1219	564 1192	5.22 7.54	3.84 1.71	48.8 21.7
0.07	50	591 1091	669 1219	636 1117	6.64 3.96	1.94 2.39	235 291
0.08	46	582	697	660	4.11	1.04	92.0
0.10	32	695	732	718	2.32	0.25	11.8
0.12	58	712	754	732	5.34	0.65	37.8

* Data for pure ZnCr_2_Se_4_, presented in Ref. [31], are shown for comparison.

## Data Availability

Not applicable.

## Supplementary Materials

The following supporting information can be downloaded at: https://www.mdpi.com/article/10.3390/ma16134565/s1. Refs. [5,6] are cited in the supplementary materials.

## References

[1] Snyder G.J., Caillat T., Fleurial J.P. (2001). Thermoelectric properties of Chalcogenides with Spinel Structure. Mat. Res. Innov..

[2] Korenskii V.I., Ignateva I.S., Kolenko I.P., Potiev A.A., Surat L.L. (1982). Osobennosti Elektronnogo Stroeniya i Svoistva Twerdofaznych Soedinenii Titana i Vanadya.

[3] Armand N.B., Minphy M.W., Broadhead J., Steele B.C.H. (1980). Materials for Advances Batteries.

[4] Thakeray M.M., David W.I.F., Goodenough J.B. (1984). High-temperature lithiation of α-Fe_2_O_3_: A mechanistic study. J. Solid State Chem..

[5] Lotgering F.K. (1965). Ferromagnetic interactions in sulphides, selenides and tellurides with spinel structure. Proceedings of the International Conference on Magnetism.

[6] Plumier R. (1966). Étude par diffraction de neutrons de l’antiferromagnétisme hélicoïdal du spinelle ZnCr_2_Se_4_ en présence d’un champ magnétique. J. Phys..

[7] Greenwood N.N. (1973). Ionenkristalle, Gitterdefekte und Nichtstöchiometrische Verbindungen.

[8] Park S., Kwon S., Lee S., Khim S., Bhoi D., Park C.B., Kim K.H. (2019). Interactions in the bond-frustrated helimagnet ZnCr_2_Se_4_ investigated by NMR. Sci. Rep..

[9] Hemberger J., von Nidda H.-A.K., Tsurkan V., Loidl A. (2007). Large Magnetostriction and Negative Thermal Expansion in the Frustrated Antiferromagnet ZnCr_2_Se_4_. Phys. Rev. Lett..

[10] Yaresko A.N. (2008). Electronic band structure and exchange coupling constants in ACr_2_X_4_ spinels (A=Zn, Cd, Hg; X=O, S, Se). Phys. Rev..

[11] Malicka E., Groń T., Pacyna A.W., Maciążek E., Duda H., Pawełczyk M., Zawisza B., Sitko R. (2009). Influence of temperature on the critical fields in ZnCr_2−x_Al_x_Se_4_ antiferromagnets. J. Alloys Compd..

[12] Groń T., Malicka E., Duda H., Pacyna A.W., Mydlarz T., Sitko R., Pawełczyk M. (2009). Spin-glass-like behavior in Zn_x_Cr_y_Al_z_Se_4_. J. Phys. Chem. Solids.

[13] Okońska-Kozłowska I., Lutz H.D., Groń T., Krok J., Mydlarz T. (1984). Darstellung, elektrische und magnetische Eingenschaften von-Zn_1−x_Ga_0.667x_Cr_2_Se_4_-Spinell-Einkristallen. Mater. Res. Bull..

[14] Groń T., Duda H., Warczewski J. (1990). Transport phenomena in the antiferromagnetic spinels Zn_1−x_Ga_2x/3_Cr_2_Se_4_ (where 0.0 < × < 0.5). J. Mag. Mag. Mater..

[15] Groń T., Kopyczok J., Okońska-Kozłowska I., Warczewski J. (1992). Seebeck effect in the antiferromagnetic single crystals of ZnCr_2−x_In_x_Se_4_ (0.0 < × < 0.15). J. Mag. Mag. Mater..

[16] Jendrzejewska I., Groń T., Kusz J., Żelechower M., Maciążek E., Ślebarski A., Fijałkowski M. (2015). Spin-glass-like behaviour in tin doped ZnCr_2_Se_4_ single crystals. J. Alloys Compd..

[17] Jendrzejewska I., Mroziński J., Groń T., Duda H., Zajdel P., Pacyna A.W., Maciążek E., Hanc A. (2009). Effect of cation substitution on Fermi level of n-type Zn_x_Sn_y_Cr_z_Se_4_ spinels. J. Alloys Compd..

[18] Jendrzejewska I., Groń T., Kusz J., Stokłosa Z., Pietrasik E., Goryczka T., Sawicki B., Goraus J., Jampilek J., Duda H. (2022). The Zn_1−x_Pb_x_Cr_2_Se_4_-single crystals obtained by chemical vapour transport—structure and magnetic, electrical, and thermal properties. Materials.

[19] Groń T., Wolff J., Hehenkamp T., Bärner K., Okońska-Kozłowska I., Jendrzejewska I., Malicka E. (1996). Positron annihilation studies in single and polycrystals of Zn_1−x_Cu_x_Cr_2_Se_4_ spinel series. Radiat. Eff. Defects Solids.

[20] Groń T., Jendrzejewska I., Gołąbek S., Duda H., Krajewski A., Bärner K. (2003). The thermoelectric power of ferromagnetically ordered Zn_x_Cu_y_Cr_z_Se_4_ single crystals. Phys. B Condens. Matter..

[21] Mazur S., Groń T., Jendrzejewska I. (2009). Influence of spin arrangement on thermopower in Zn_x_Cu_y_Cr_z_Se_4_ spinels. J. Alloys Compd..

[22] Jendrzejewska I., Waśkowska A., Mydlarz T. (2001). Influence of nickel substitution on the cation distribution and magnetic properties of ZnCr_2_Se_4_. J. Alloys Compd..

[23] Jendrzejewska I., Zajdel P., Goryczka T., Goraus J., Kita A., Mydlarz T. (2012). Influence of covalency and anion polarisation on magnetic and electronic properties of ZnCr_2−x_Ni_x_Se_4_. J. Alloys Compd..

[24] Jendrzejewska I., Groń T., Goraus J., Pilch M., Pietrasik E., Barsova Z., Czerniewski J., Goryczka T., Witkowska-Kita B., Bienko A. (2019). Synthesis and structural, magnetic, thermal and electronic properties of Mn-doped ZnCr_2_Se_4_. Mater. Chem. Phys..

[25] Jendrzejewska I., Zajdel P., Heimann J., Krok-Kowalski J., Mydlarz T., Mrzigod J. (2012). Influence of manganese on magnetic and electronic properties of ZnCr_2_Se_4_ single Crystals. Mater. Res. Bull..

[26] Jendrzejewska I., Groń T., Kwapuliński P., Kusz J., Pietrasik E., Goryczka T., Sawicki B., Ślebarski A., Fijałkowski M., Jampilek J. (2021). Study of the structure, magnetic, thermal and electrical characterisation of ZnCr_2_Se_4_:Ta single crystals obtained by chemical vapour transport. Materials.

[27] Jendrzejewska I., Groń T., Knizek K., Pilch M., Ślebarski A., Goraus J., Zajdel P., Stokłosa Z., Pietrasik E., Goryczka T. (2021). Preparation, structure and magnetic, electronic and thermal properties of Dy^3+^-doped ZnC_r2_S_e4_ with unique geometric type spin-glass. J. Solid State Chem..

[28] Jendrzejewska I., Groń T., Maciążek E., Duda H., Kubisztal M., Ślebarski A., Pietrasik E., Fijałkowski M. (2016). Specific heat and magnetic properties of single-crystalline Zn_x_Dy_y_Cr_z_Se_4_ spinels. J. Magn. Magn. Mater..

[29] Maciążek E., Karolus M., Kubisztal M., Jendrzejewska I., Sitko R., Groń T., Ślebarski A., Fijałkowski M. (2015). Magnetic and specific heat properties of a new Gd-doped ZnCr_2_Se_4_. Mater. Chem. Phys..

[30] Jendrzejewska I., Groń T., Kusz J., Goraus J., Barsova Z., Pietrasik E., Czerniewski J., Goryczka T., Kubisztal M. (2020). Growth, structure and physico-chemical properties of monocrystalline ZnCr_2_Se_4_:Ho prepared by chemical vapour transport. J. Solid State Chem..

[31] Jendrzejewska I., Groń T., Kusz J., Barsova Z., Pietrasik E., Goryczka T., Sawicki B., Ślebarski A., Fijałkowski M., Jampilek J. (2020). Synthesis, crystal structure and characterisation of monocrystalline ZnCr_2_Se_4_ doped with neodymium. J. Solid State Chem..

[32] Piekarczyk J. (1987). Thermodynamic model of chemical vapour transport and its application to some ternary compounds: I. The model. J. Cryst. Growth.

[33] Piekarczyk J. (1988). Thermodynamic model of chemical vapour transport and its application to some ternary compounds: II. Application of the model to the complex oxides: ZnCr_2_O_4_, Y_3_Fe_5_O_12_ and Fe_2_TiO_5_. J. Cryst. Growth.

[34] Schmidt P.M., Binnewies M., Glaum R.M., Schmidt M. (2013). Chemical Vapor Transport Reactions—Methods, Materials, Modeling. Advanced Topics on Crystal Growth.

[35] Jendrzejewska I., Żelechower M., Szamocka K., Mydlarz T., Waśkowska A., Okońska-Kozłowska I. (2004). Growth of the Cd_x_Ni_y_Cr_z_Se_4_ single crystals and their magnetic properties. J. Cryst. Growth.

[36] (2009). HSC Chemistry.

[37] Jurczyk J., Sitko R., Jendrzejewska I. (1999). Thin sample in the XRF analysis. A new method of preparing microsamples of mono- and polycrystals, and silicate rocks. Chem. Anal..

[38] Groń T., Krok-Kowalski J., Duda H., Mydlarz T., Gilewski A., Walczak J., Filipek E., Bärner K. (1995). Metamagnetism in Cr_2_V_4−*x*_Mo*_x_*O_13+0.5*x*_. Phys. Rev. B.

[39] Krok-Kowalski J., Groń T., Warczewski J., Mydlarz T., Okońska-Kozłowska I. (1997). Ferrimagnetism and metamagnetism in Cd_1−x_Cu_x_Cr_2_S_4_, spinels. J. Magn. Magn. Mater..

[40] Morrish A.H. (1965). Physical Principles of Magnetism.

[41] Workman R.L., Burkert V.D., Crede V., Klempt E., Thoma U., Tiator L., Agashe K., Aielli G., Allanach B.C., Particle Data Group (2022). The Review of Particle Physics. Prog. Theor. Exp. Phys..

[42] Holland W.E., Brown H.A. (1972). Application of the Weiss molecular field theory to the B-site spinel. Phys. Status Solidi.

[43] Clark R.C., Reid J.S. (1995). The analytical calculation of absorption in multifaceted crystals. Acta Crystallogr..

[44] Sheldrick G.M. (2008). A short history of SHELX. Acta Crystallogr..

[45] Plies V. (1988). Massenspektrometrische Untersuchungen der Gasphase über CrCl_3_ und CrCl_3_/Cl_2_. Z. Anorg. Allg. Chem..

[46] Von Oppermann H. (1968). Das Reaktionsgleichgewicht 2CrCl_3_,f, g + Cl_2_,g = 2 CrCl_4_,g. Z. Anorg. Allg. Chem..

[47] Shannon R.D. (1976). Revised Effective Ionic Radii and Systematic Studies of Interatomic Distances in Halides and Chalcogenides. Acta Cryst..

[48] Dzięgielewski J. (1987). Inorganic Chemistry—Script for Students.

[49] Busch G., Magyar B., Wachter P. (1966). Optical absorption of some ferro- and antiferromagnetic spinels, containing Cr^3+^-ions. Phys. Lett..

[50] Barnard R.D. (1972). Thermoelectricity in Metals and Alloys.

[51] Trodahl H.J. (1995). Thermopower of the superconducting cuprates. Phys. Rev. B.

[52] Kittel C. (1971). Introduction to Solid State Physics.

[53] Groń T., Bärner K., Kleeberg C., Okońska-Kozłowska I. (1996). The thermoelectric power of ferromagnetically ordered Cu_1−*x*_Ga*_x_*Cr_2_Se_4_ spinels. Phys. B Condens. Matter.

[54] Sawicki B., Karolewicz M., Tomaszewicz E., Oboz M., Groń T., Kukuła Z., Pawlus S., Nowok A., Duda H. (2021). Effect of Gd^3+^ substitution on thermoelectric power factor of paramagnetic Co^2+^-doped calcium molybdato-tungstates. Materials.

[55] Sawicki B., Tomaszewicz E., Guzik M., Groń T., Oboz M., Duda H., Pawlus S., Urbanowicz P. (2023). Effect of Ca^2+^ site substitution on structural, optical, electrical and magnetic properties in Nd^3+^ and Mn^2+^-co-doped calcium molybdato-tungstates. Ceram. Int..

[56] Malicka E., Groń T., Ślebarski A., Pacyna A.W., Goraus J., Fijałkowski M., Heimann J. (2011). Specific heat and magnetic susceptibility of single-crystalline ZnCr_2−x_Al_x_Se_4_ (x = 0.15, 0.23). J. Phys. Chem. Solids.

[57] Malicka E., Groń T., Ślebarski A., Gągor A., Pacyna A.W., Sitko R., Goraus J., Mydlarz T., Heimann J. (2011). Specific heat and magnetic susceptibility of single-crystalline ZnCr_2_Se_4_ spinels doped with Ga, In and Ce. Mater. Chem. Phys..

