# Supplementary material for: Synthesis, Structure, and Physicochemical Characteristics of Zn1−xRexCr2Se4 Single Crystals
Source: Materials (Basel). 2023 Jun 24;16(13):4565. doi: 10.3390/ma16134565 (PMC10342625; doi:10.3390/ma16134565)
Supplement: Supplementary file 1 [file materials-16-04565-s001.zip › Supplementary Materials.pdf]

Supplementary materials

Synthesis, Structure, and Physicochemical Characteristics of $Zn_{1-x}Re_xCr_2Se_4$ Single Crystals

Izabela Jendrzejewska [1,*], Tadeusz Groń [2], Joachim Kusz [2], Zbigniew Stokłosa [3], Ewa Pietrasik [1], Tomasz Goryczka [3], Bogdan Sawicki [2], Jerzy Goraus [2], Josef Jampilek [4,*] and Beata Witkowska-Kita [5]

The set of these reactions was used to prepare the thermodynamic model of crystal growth.

1. $2ZnSe + 2CrCl_{3(g)} = 2ZnCl_{(g)} + 2CrCl_{2(g)} + Se_{2(g)}$
2. $2ZnSe + 2CrCl_{3(g)} = ZnCl_{2(g)} + 2CrCl_{2(g)} + Se_{2(g)}$
3. $Re + 2CrCl_{3(g)} = ReCl_{2(g)} + 2CrCl_{2(g)}$
4. $Se + 2CrCl_{3(g)} = SeCl_{2(g)} + 2CrCl_{2(g)}$
5. $Se + 4CrCl_{3(g)} = SeCl_{4(g)} + 4CrCl_{2(g)}$
6. $2ZnSe + 2CrCl_{4(g)} = 2ZnCl_{(g)} + 2CrCl_{2(g)} + Se_{2(g)}$
7. $2ZnSe + 2CrCl_{4(g)} = 2ZnCl_{2(g)} + 2CrCl_{2(g)} + Se_{2(g)}$
8. $2Re + 2CrCl_{4(g)} = 2ReCl_{2(g)} + 2CrCl_{2(g)}$
9. $Se + CrCl_{4(g)} = SeCl_{2(g)} + CrCl_{2(g)}$
10. $Se + CrCl_{4(g)} = SeCl_{4(g)} + CrCl_{2(g)}$
11. $2ZnSe + Cl_{2(g)} = 2ZnCl_{(g)} + Se_{2(g)}$
12. $2ZnSe + 2Cl_{2(g)} = 2ZnCl_{2(g)} + Se_{2(g)}$
13. $2Re + 4Cl_{2(g)} = 2ReCl_{2(g)} + 2Cl_{2(g)}$
14. $Se + Cl_{2(g)} = SeCl_{2(g)}$
15. $Se + 2Cl_{2(g)} = SeCl_{4(g)}$

Table S1. Crystal data and structure refinement of the $ZnCr_2Se_4$: Re single crystals.

Crystal data	(1)	(2)	(3)	(4)	(5)
Chemical formula	$(Zn_{0.96}Re_{0.06})Cr_2Se_4$	$(Zn_{0.93}Re_{0.07})Cr_2Se$	$(Zn_{0.92}Re_{0.08})Cr_2Se_4$	$(Zn_{0.90}Re_{0.10})Cr_2Se_4$	$(Zn_{0.88}Re_{0.12})Cr_2Se_4$
Temperature (K)	293	293	293	293	293
Crystal system	Cubic	Cubic	Cubic	Cubic	Cubic
Space group	$Fd\bar{3}m$	$Fd\bar{3}m$	$Fd\bar{3}m$	$Fd\bar{3}m$	$Fd\bar{3}m$
Z	8	8	8	8	8
a (Å)	10.49202(19)	10.49226(16)	10.49448(14)	10.49635(14)	10.50025(15)
Volume (Å^3)	1155.01(5)	1155.00(3)	1155.80(1)	1156.42(2)	1157.71(1)
Density calc.(Mg/m^3)	5.664	5.678	5.688	5.713	5.734
Absorption coeff.(mm^{-1})	33.879	34.048	34.192	34.509	34.806
Radiation, wavelength (Å)	Mo, K_α, 0.71073				
Limiting indices *h*	-18, 17	-19, 17	-14, 19	-17, 19	-18,18

k	-18, 14	-18, 14	-18, 19	-19, 15	-18,15
l	-18, 18	-18, 18	-19, 18	-19, 19	-14,19
Absorption correction	multi - scan				
F(000)	1734	1737	1741	1748	1755
Crystal size (mm^3)	0.060 x 0.040 x 0.010	0.140 x 0.080 x 0.010	0.100 x 0.080 x 0.030	0.120 x 0.040 x 0.010	0.100 x 0.060 x 0.020
Theta range for data collection	3.363 to 40.787°	3.363 to 40.721°	3.362 to 40.244°	3.362 to 40.701°	3.361 to 40.683°
Reflections collected	5194	5143	5190	5203	5197
Independent reflections	213 [R(int) = 0.0402]	213 [R(int) = 0.0440]	211 [R(int) = 0.0314]	212 [R(int) = 0.0361]	213 [R(int) = 0.0362]
Completeness to theta = 25.242°	100%	100%	100%	100%	100%
Refinement					
Refinement method	Full-matrix least-squares on F^2				
Data/restraints/parameters	212/0/9	213/0/9	211/0/8	212/0/8	213/0/7
Number of refined parameters	8	8	8	8	8
Goodness of fit on F^2	1.176	1.172	1.182	1.212	1.169
Final R indices [I>2σ(I)]	R1 = 0.0236, wR2 = 0.0710	R1 = 0.0245, wR2 = 0.0760	R1 = 0.0266, wR2 = 0.0882	R1 = 0.0326, wR2 = 0.1077	R1 = 0.0413, wR2 = 0.1259
R indices (all data)	R1 = 0.0242, wR2 = 0.0720	R1 = 0.0248, wR2 = 0.0764	R1 = 0.0266, wR2 = 0.0882	R1 = 0.0327, wR2 = 0.1078	R1 = 0.0417, wR2 = 0.1263
Extinction coefficient	0.00194(19)	0.00060(11)	0.00032(11)	0.0011(2)	n/a
Largest diff. peak and hole	1.837 and -3.464 e.$Å^{-3}$	2.215 and -4.415 e.$Å^{-3}$	2.652 and -5.220 e.$Å^{-3}$	3.089 and -6.382 e.$Å^{-3}$	2.156 and -7.757 e.$Å^{-3}$

Table S2 presents the determined values of parameter *u*, atomic coordinates, and equivalent isotropic displacement parameters of the $Zn_{1-x}Re_xCr_2Se_4$ single crystals. The parameter *u*, defining the anion sublattice distortion from the cubic-close packing, possesses a value slightly different from the parent compound $ZnCr_2Se_4$. The ideal value of *u* for pure $ZnCr_2Se_4$ is 0.259 [1,2]. In Table S3, the selected bond distances and angles are shown.

1. Lotgering, F. K. In Proceedings of the International Conference on Magnetism, Nottingham, 1964, p. 533 (Institute of Physics and the Physical Society, London, 1965).
2. Plumier, R. Étude par diffraction de neutrons de l'antiferromagnétisme hélicoïdal du spinelle $ZnCr_2Se_4$ en présence d'un champ magnétique. *J. de Phys.* **1966**, 27, 213–219.

Table S2. Atomic coordinates and equivalent isotropic displacement parameters of the $Zn_{1-x}Re_xCr_2Se_4$ single crystals.

Spinel (XRD composition)	Anion parameter *u*	Site occupation (A)	[B]	U_{iso} (Å×10³) Zn/Re	Cr	Se
$(Zn_{0.94}Re_{0.06})[Cr_{2.0}]Se_{4.0}$	0.2595(1)	0.94(1):0.06(1)	1.0	14(1)	7(1)	7(1)
$(Zn_{0.93}Re_{0.07})[Cr_{2.0}]Se_{4.0}$	0.2595(1)	0.93(2):0.07(1)	1.0	14(1)	7(1)	7(1)
$(Zn_{0.92}Re_{0.08})[Cr_{2.0}]Se_{4.0}$	0.2595(1)	0.92(1):0.08(1)	1.0	13(1)	6(1)	6(1)
$(Zn_{0.90}Re_{0.10})[Cr_{2.0}]Se_{4.0}$	0.2595(1)	0.90(2):0.10(2)	1.0	15(1)	6(1)	7(1)
$(Zn_{0.88}Re_{0.12})[Cr_{2.0}]Se_{4.0}$	0.2595(1)	0.88(1):0.11(2)	1.0	15(1)	6(1)	6(1)

The Wyckoff positions of the atoms in the spinel structure are Zn/Re in 8 *b* (3/8, 3/8, 3/8), Cr in 16*c* (½, ½, ½) and Se in 32 *e* (x, x, x).

Table S3. Selected interatomic distances (Å) and bond angles (deg) of the $Zn_{1-x}Re_xCr_2Se_4$ single crystals.

Spinel	Bond distances Zn/Re–Se	Cr/–Se	Bond angles Zn–Se/Re–Se	Se–Cr–Se
$(Zn_{0.94}Re_{0.06})[Cr_2]Se_4$	2.4440(5)	2.5274(3)	109.5(0)×6	180.0(0)×3<br>94.598(13)×6<br>85.402(13)×6
$(Zn_{0.93}Re_{0.07})[Cr_2]Se_4$	2.4441(5)	2.5274(3)	109.5(0)×6	180.0(0)×3<br>94.602(2)×6<br>85.398(2)×6
$(Zn_{0.92}Re_{0.08})[Cr_2]Se_4$	2.4449(6)	2.5278(3)	109.5(0)×6	180.0(0)×3<br>94.610(14)×6<br>85.390(14)×6
$(Zn_{0.90}Re_{0.10})[Cr_2]Se_4$	2.4458(7)	2.5280(4)	109.5(0)×6	180.0(0)×3<br>94.622(2)×6<br>82.378(2)×6
$(Zn_{0.88}Re_{0.12})[Cr_2]Se_4$	2.4468(8)	2.5289(4)	109.5(0)×6	180.0(0)×3<br>94.630(2)×6<br>85.370(2)×6
